# Crystal structure of [1,3-bis­(2,4,6-tri­methyl­phen­yl)imidazolidin-2-yl­idene]di­chlorido­{2-[1-(di­methyl­amino)­eth­yl]benzyl­idene}ruthenium including an unknown solvate

**DOI:** 10.1107/S2056989019001725

**Published:** 2019-02-08

**Authors:** Kirill B. Polyanskii, Kseniia A. Alekseeva, Pavel A. Kumandin, Zeliha Atioğlu, Mehmet Akkurt, Flavien A. A. Toze

**Affiliations:** a Organic Chemistry Department, Faculty of Science, Peoples’ Friendship University of Russia (RUDN University), 6 Miklukho-Maklaya St., Moscow 117198, Russian Federation; b İlke Education and Health Foundation, Cappadocia University, Cappadocia Vocational College, The Medical Imaging Techniques Program, 50420 Mustafapaşa, Ürgüp, Nevşehir, Turkey; c Department of Physics, Faculty of Sciences, Erciyes University, 38039 Kayseri, Turkey; d Department of Chemistry, Faculty of Sciences, University of Douala, PO Box 24157, Douala, Republic of Cameroon

**Keywords:** crystal structure, ruthenium catalyst, Hoveyda–Grubbs catalyst, N—Ru coordination bond, metathesis of olefins, SQUEEZE

## Abstract

The title compound, [RuCl_2_(C_21_H_26_N_2_)(C_11_H_15_N)], is an example of a new generation of *N*,*N*-dialkyl metallocomplex ruthenium catalysts with an N→Ru coordination bond as part of a six-membered chelate ring. The Ru atom has an Addison τ parameter of 0.234, which indicates a geometry inter­mediate between square-based pyramidal and trigonal–bipyramidal. The complex shows the usual *trans* arrangement of the two chloride ligands, with Ru—Cl bond lengths of 2.3397 (8) and 2.3476 (8) Å, and a Cl—Ru— Cl angle of 157.47 (3)°. The crystal structure features C—H⋯Cl, C—H⋯π and π–π stacking inter­actions. The solvent mol­ecules were found to be highly disordered and their contribution to the scattering was removed with the SQUEEZE procedure in *PLATON* [Spek (2015). *Acta Cryst.* C**71**, 9–18], which indicated a solvent cavity of volume 1096 Å^3^ containing approximately 419 electrons. These solvent mol­ecules are not considered in the given chemical formula and other crystal data.

## Abstract 2

The compound [RuCl_2_(C_21_H_26_N_2_)(C_11_H_15_N)] is an example of a new generation of *N*,*N*-dialkyl metallocomplex ruthenium catalysts with an N→Ru coordination bond in a six-membered chelate ring.

## Chemical context   

Since the 1980s metathesis has become an important industrial process, but applications of the first-generation catalysts to targets bearing various functional groups were often precluded by the dramatic increase of their catalytic activity (Delaude & Noels, 2005 ▸; Astruc, 2005 ▸). Hence, in recent years a large number of new catalysts have been proposed, developed and implemented in organic chemistry processes. These new catalysts may be used in the presence of various functional groups, moisture traces, in a wide range of solvents under different temperatures and for many metathesis reactions including CM (cross metathesis), ROM (ring-opening metathesis), RCM (ring-closing metathesis), ROMP (ring-opening metathesis polymerization), ADMET (acyclic diene metathesis polymerization) and others (Dragutan *et al.*, 2005 ▸; Grubbs *et al.*, 2015 ▸; Hoveyda & Zhugralin, 2007 ▸). Currently, the most widely used catalysts are ruthenium-based heterocyclic carbene-coordinated metallocomplexes, containing, as rule, a five-membered ruthenium-containing ring with an *O*→Ru coordination bond (the Hoveyda–Grubbs catalysts of the second generation) (Ogba *et al.*, 2018 ▸; Samojłowicz & Grela, 2009 ▸; Vougioukalakis & Grubbs, 2010 ▸).

Currently, there is only scarce information about the synthesis and application in the metathesis reactions of the nitro­gen-containing Grubbs catalysts, where the oxygen atom is substituted by an N atom in a five-membered ring. The known compounds of that type have promising catalytic properties and are already used in the industry. For example, there is patent information that describes applications of such a type of catalysts in ring-opening metathesis polymerization reactions (Zheng-Yun, 2017 ▸; Xia, 2017 ▸; Zheng-Yun, 2011 ▸; Polyanskii *et al.*, 2015 ▸; Ivin & Mol, 1997 ▸).
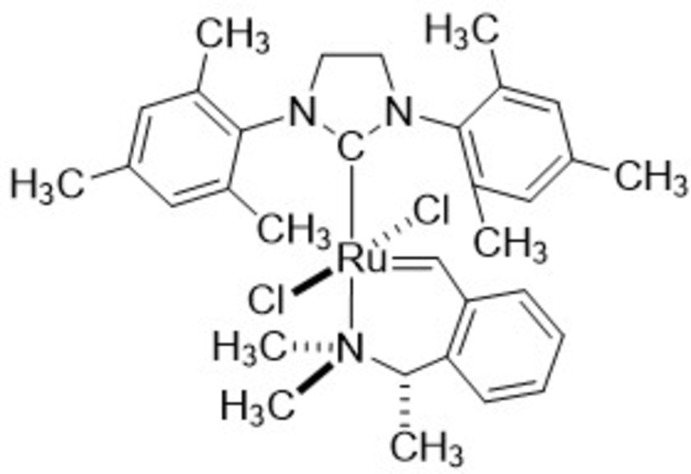


The purpose of this study is to elaborate the synthesis of new generation of *N*,*N*-dialkyl metallocomplex ruthenium catalysts, resulting in establishment of connection between the nature of the functional groups born by the nitro­gen atom and the catalytic activity and stability of these catalysts in various metathesis reactions as well as in the determination of the effect of substituents on the structures of the obtained products.

## Structural commentary   

**Figure 1:**
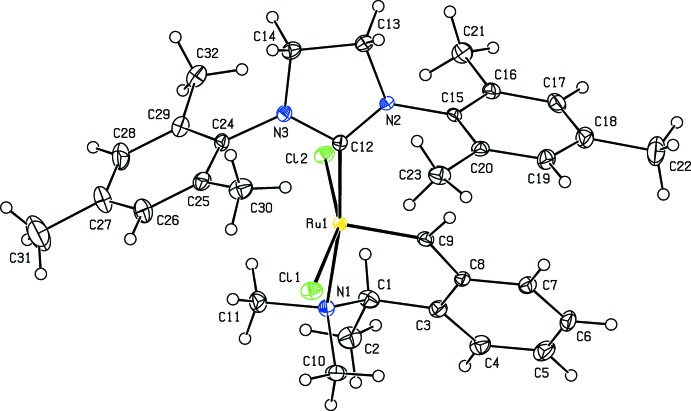
The mol­ecular structure of the title complex with displacement ellipsoids for the non-hydrogen atoms drawn at the 50% probability level.

The Ru atom in the title compound is penta­coordinated to two C, one N and two Cl atoms (Table 1 ▸). The Addison τ parameter is used to describe the distortion of the coordination geometry and is defined as τ = (difference between two largest angles/60) for five-coordinated metal centers, allowing the distinction between trigonal–bipyramidal (ideally τ = 1) and square-pyramidal (ideally τ = 0) geometries. For the title complex, τ = 0.234, in between these two geometries (Figs. 1 ▸ and 2 ▸). The dihedral angle between the planes of the tri­methyl­phenyl rings is 31.95 (19)°. The complex shows the usual *trans* arrangement of the two chloride ligands, with Ru—Cl bond lengths of 2.3397 (8) and 2.3476 (8) Å, and a Cl—Ru— Cl angle of 157.47 (3)°. The bond lengths and angles about the Ru atom are in good agreement with those in the di­chloro­methane solvate [(SPY-5-34)-di­chloro­(2-formyl­benzyl­idene-κ^2^-*C*,*O*)[1,3-bis­(2,4,6-tri­methyl­phen­yl)-4,5-di­hydro­imidazol-2-yl­idene]ruthenium] (Slugovc *et al.*, 2004 ▸) and *cis*-di­chlorido­(1,3-dimesitylimidazolidin-2-yl­idene)(2-formyl­benzyl­idene-κ^2^ *C*,*O*)ruthenium diethyl ether solvate (Slugovc *et al.*, 2010 ▸).

## Supra­molecular features   

**Figure 3:**
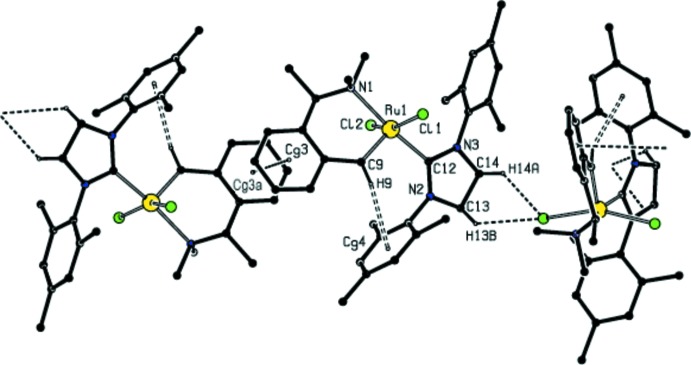
A partial packing diagram of the title compound, showing the C—H⋯Cl, C—H⋯π and π–π stacking inter­actions as dashed lines [symmetry code: (a) 1 − *x*, −*y*, 1 − *z*].

The crystal structure features C—H⋯Cl, C—H⋯π inter­actions (Table 2 ▸) and π–π stacking inter­actions between the benzyl­idene rings [centroid–centroid distance = 3.684 (3) Å, inter-planar distance = 3.5312 (16) Å and slippage = 1.048 Å], forming a three-dimensional network. The hydrogen-bonding inter­actions in the title complex are shown in Fig. 3 ▸.

## Database survey   

Both *cis*-di­chlorido­(1,3-dimesitylimidazolidin-2-yl­idene)(2-formyl­benzyl­idene-κ^2^ *C*,*O*)ruthenium diethyl ether solvate (Slugovc *et al.*, 2010 ▸) and the di­chloro­methane solvate [(SPY-5-34)-di­chloro­(2-formyl­benzyl­idene-κ^2^ *C*,*O*)[1,3-bis­(2,4,6-tri­methyl­phen­yl)-4,5-di­hydro­imidazol-2-yl­idene]ruthenium] (Slugovc *et al.*, 2004 ▸), show similar metal-atom geometries to the title compound. In contrast to the di­chloro­methane solvate, where the Ru complexes do not show any inter­molecular π–π-stacking but are linked by C—H⋯π and C—H⋯Cl inter­actions (Jlassi *et al.*, 2014 ▸; Ma *et al.*, 2017*a* ▸,*b* ▸; Shixaliyev *et al.*, 2013 ▸, 2014 ▸, 2018 ▸), inter­molecular π–π stacking is an important factor in the crystal structures of the title complex and *cis*-di­chlorido­(1,3-dimesitylimidazolidin-2-yl­idene)(2-formyl­benzyl­idene-κ^2^ *C*,*O*)ruthenium diethyl ether solvate where these inter­actions form a framework-like structure containing channels that extend along the *b* and *c* axes, respectively. The crystal structures of some ruthenium-based heterocyclic carbene-coordinated metallo-complexes, containing a five-membered ruthenium-containing cycle with an *O*→Ru coordination bond have been reported by Samojłowicz *et al.* (2009 ▸).

## Synthesis and crystallization   

**Figure 4:**
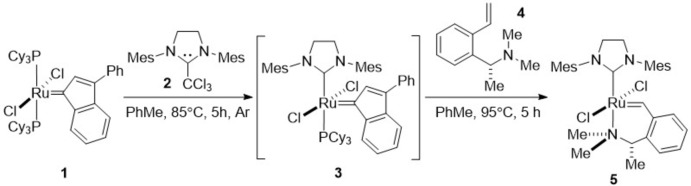
Reaction scheme.

The synthesis of the title complex (**5**) was performed by the inter­action of the indenyl­idene derivative (**1**) with 1,3-dimes­ityl-2-(tri­chloro­meth­yl)imidazolidine (**2**). The inter­mediate (**3**), which is unstable in air, was not isolated and was directed to the following reaction step with styrene (**4**) as described earlier (Dorta *et al.*, 2004 ▸; Fürstner *et al.*, 2001 ▸; Jimenez *et al.*, 2012 ▸; Pump *et al.*, 2015 ▸) (Fig. 4 ▸). The catalyst (**5**) was obtained in moderate yield and turned out to be a green powder, stable in air at room temperature for at least four years.

**Synthesis of the Hoveyda–Grubbs catalyst (5)**:

Absolute toluene (50 ml), di­chloro­(3-phenyl-1*H*-inden-1-yl­idene)bis­(tri­cyclo­hexyl­phosphane)ruthenate (**1**) (3.52 g, 3.81 mmol) and 1,3-bis­(2,4,6-tri­methyl­phen­yl)-2-tri­chloro­methyl­imidazolidine (**2**) (1.94 g, 4.56 mmol) were placed into a 100 ml Schlenk flask purged with argon. The mixture was heated under argon at 358 K for 5 h, then the mixture was cooled at room temperature and 1-(2-ethenylphen­yl)-*N*,*N*-di­methyl­ethanamine (**4**) (1.00 g, 5.71 mmol) was added under an argon atmosphere. The mixture was heated under argon at 368 K for 5 h. Toluene was evaporated under reduced pressure and the residue was suspended in hexane (30 ml). The resulting mixture was kept at 253 K for 10 h. The obtained precipitate was filtered off, washed with hexane (3 × 10 ml) and methanol (2 × 10ml), and dried under vacuum at room temperature to give 1.90 g (2.96 mmol, yield 79%) of **5** as a light-green powder, pure by TLC, m.p 455–458 K (decomp.). Green prisms were grown by slow crystallization from a hepta­ne–CH_2_Cl_2_ solvent mixture.

^1^H NMR (500.1 MHz, CD_2_Cl_2_, 571 K) δ, ppm: 18.74 (*s*, 1H, CH=Ru), 7.58 (*dt*, *J* = 1.3 and *J* = 7.7 Hz, 1H, H-3-C_6_H_4_), 7.24 (*br d*, *J* = 7.7 Hz, 1H, H-4—C_6_H_4_), 7.22 (*t*, *J* = 7.7 Hz, 1H, H-5—C_6_H_4_), 7.11 (*br s*, 2H, H—Mes), 7.04 (*br s*, 2H, H—Mes), 6.76 (*d*, *J* = 7.7 Hz, 1H, H-2—C_6_H_4_), 5.74 (*q*, *J* = 6.7 Hz 1H, N—CH—Me), 4.11 (*very br s*, 4H, N—CH_2_—CH_2_—N), 2.56 (*very br s*, 12H, Me—Mes), 2.43 (*s*, 6H, Me—Mes), 2.05 (*s*, 3H, NMe), 1.53 (*s*, 3H, NMe), 1.39 (*d*, *J* = 6.7 Hz, 3H, CHMe). ^13^C NMR (125.7 MHz, CD_2_Cl_2_, 571 K) δ, ppm: 312.3 (C=Ru), 213.0 (N—C—N), 148.7 (C-6—C_6_H_4_), 138.4 (8C, *br s*, C—Mes), 137.2 (C-1—C_6_H_4_), 129.3 (*very br s*, 4C, CH—Mes), 129.0 (C-5—C_6_H_4_), 128.4 (C-4—C_6_H_4_), 128.3 (C-3—C_6_H_4_), 127.0 (C-2—C_6_H_4_), 59.0 (NCH—Me), 51.5 and 50.1 (NCH_2_CH_2_N), 43.2 (NMe), 38.5 (NMe), 20.8 (6C, Me—Mes), 9.6 (NCH—Me). IR ν_max_/cm^−1^ (KBr): 2953, 2915, 1605, 1481, 1443, 1377, 1256, 1183, 1117, 1041, 848, 806, 779, 578. HR–MALDI–ToF MS: 604.20 [*M* − Cl]^+^. Analysis calculated for C_32_H_41_Cl_2_N_3_Ru: C 60.09, H 6.46, N 6.57%. Found: C 59.83, H 6.24, N 6.92%.

## Refinement   

Crystal data, data collection and structure refinement details are summarized in Table 3 ▸. C-bound H atoms were included in the refinement using the riding-model approximation with C—H distances of 0.93–0.97 Å, and with *U*_iso_(H) = 1.2 or 1.5*U*_eq_(C). The measurements of the 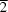
02, 002), 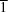
11, 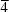
02, 110, 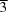
12), 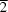
21, 200 and 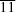
35 reflections were affected by shielding by the beam stop and were therefore excluded from the refinement. A region of electron density, most probably disordered solvent mol­ecules, occupying voids of *ca* 1096 Å^3^ for an electron count of 419, was removed with the SQUEEZE procedure in *PLATON* (Spek, 2015 ▸) following unsuccessful attempts to model it as a plausible solvent mol­ecule. The stated formula mass, density, *etc*. do not include the disordered solvent.

## Supplementary Material

Crystal structure: contains datablock(s) I. DOI: 10.1107/S2056989019001725/hb7792sup1.cif

Structure factors: contains datablock(s) I. DOI: 10.1107/S2056989019001725/hb7792Isup2.hkl

CCDC reference: 1894660

Additional supporting information:  crystallographic information; 3D view; checkCIF report

## Figures and Tables

**Figure 2:**
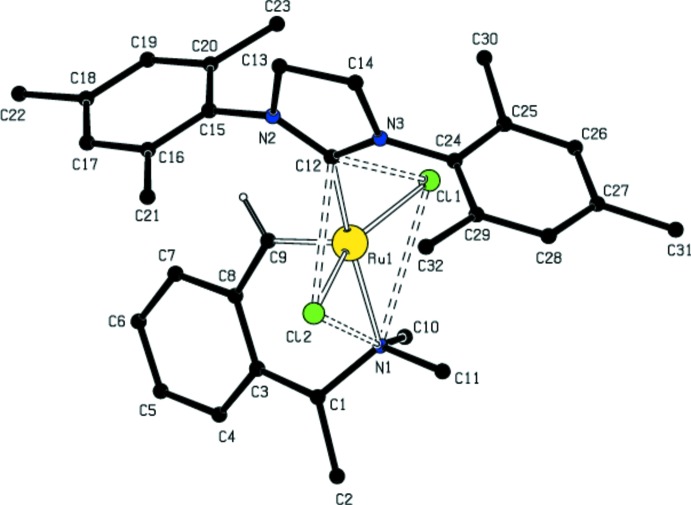
A view of the coordination geometry about the Ru atom, which lies between square-based pyramidal and trigonal–bipyramidal.

**Table 1:** Selected geometric parameters (Å, °)

Ru1—C9	1.822 (3)	Ru1—Cl1	2.3397 (8)
Ru1—C12	2.036 (3)	Ru1—Cl2	2.3476 (8)
Ru1—N1	2.265 (3)		
			
C9—Ru1—C12	99.91 (12)	N1—Ru1—Cl1	88.15 (7)
C9—Ru1—N1	87.49 (12)	C9—Ru1—Cl2	101.93 (10)
C12—Ru1—N1	171.53 (10)	C12—Ru1—Cl2	85.25 (8)
C9—Ru1—Cl1	100.30 (10)	N1—Ru1—Cl2	89.21 (7)
C12—Ru1—Cl1	94.51 (8)	Cl1—Ru1—Cl2	157.47 (3)

**Table 2:** Hydrogen-bond geometry (Å, °)

*D*—H⋯*A*	*D*—H	H⋯*A*	*D*⋯*A*	*D*—H⋯*A*
C13—H13*B*⋯Cl1^i^	0.97	2.82	3.578 (3)	135
C14—H14*A*⋯Cl1^i^	0.97	2.82	3.576 (4)	135
C9—H9⋯*Cg*4	0.93	2.61	3.481 (4)	157

Symmetry code: (i) 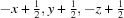.

**Table 3:** Experimental details

Crystal data	
Chemical formula	[RuCl_2_(C_21_H_26_N_2_)(C_11_H_15_N)]
*M*_r_	639.65
Crystal system, space group	Monoclinic, *C*2/*c*
Temperature (K)	296
*a*, *b*, *c* (Å)	35.8175 (17), 10.5633 (5), 24.0946 (11)
β (°)	131.781 (2)
*V* (Å^3^)	6797.9 (6)
*Z*	8
Radiation type	Mo *K*α
μ (mm^−1^)	0.64
Crystal size (mm)	0.34 × 0.28 × 0.21

Data collection	
Diffractometer	Bruker APEXII CCD
No. of measured, independent and observed [*I* > 2σ(*I*)] reflections	21100, 7485, 5440
*R*_int_	0.041
(sin θ/λ)_max_ (Å^−1^)	0.642

Refinement	
*R*[*F* ^2^ > 2σ(*F* ^2^)], *wR*(*F* ^2^), *S*	0.038, 0.110, 1.04
No. of reflections	7485
No. of parameters	352
H-atom treatment	H-atom parameters constrained
Δρ_max_, Δρ_min_ (e Å^−3^)	0.46, −0.44

Computer programs: *APEX2* and *SAINT* (Bruker, 2007 ▸), *SHELXS97* (Sheldrick, 2008 ▸), *SHELXL2014* (Sheldrick, 2015 ▸), *ORTEP-3 for Windows* (Farrugia, 2012 ▸) and *PLATON* (Spek, 2009 ▸).

## supplementary crystallographic information

### Crystal data

**Table:**

[RuCl_2_(C_21_H_26_N_2_)(C_11_H_15_N)]	*F*(000) = 2656
*M**_r_* = 639.65	*D*_x_ = 1.250 Mg m^−^^3^
Monoclinic, *C*2/*c*	Mo *K*α radiation, λ = 0.71073 Å
*a* = 35.8175 (17) Å	Cell parameters from 4879 reflections
*b* = 10.5633 (5) Å	θ = 2.6–24.9°
*c* = 24.0946 (11) Å	µ = 0.64 mm^−^^1^
β = 131.781 (2)°	*T* = 296 K
*V* = 6797.9 (6) Å^3^	Block, green
*Z* = 8	0.34 × 0.28 × 0.21 mm

### Data collection

**Table:**

Bruker APEXII CCD diffractometer	*R*_int_ = 0.041
φ and ω scans	θ_max_ = 27.1°, θ_min_ = 3.0°
21100 measured reflections	*h* = −45→45
7485 independent reflections	*k* = −13→12
5440 reflections with *I* > 2σ(*I*)	*l* = −29→30

### Refinement

**Table:**

Refinement on *F*^2^	Primary atom site location: structure-invariant direct methods
Least-squares matrix: full	Hydrogen site location: inferred from neighbouring sites
*R*[*F*^2^ > 2σ(*F*^2^)] = 0.038	H-atom parameters constrained
*wR*(*F*^2^) = 0.110	*w* = 1/[σ^2^(*F*_o_^2^) + (0.0547*P*)^2^ + 3.4409*P*] where *P* = (*F*_o_^2^ + 2*F*_c_^2^)/3
*S* = 1.04	(Δ/σ)_max_ < 0.001
7485 reflections	Δρ_max_ = 0.46 e Å^−^^3^
352 parameters	Δρ_min_ = −0.44 e Å^−^^3^
0 restraints	

### Special details

**Table:**

Geometry. All esds (except the esd in the dihedral angle between two l.s. planes) are estimated using the full covariance matrix. The cell esds are taken into account individually in the estimation of esds in distances, angles and torsion angles; correlations between esds in cell parameters are only used when they are defined by crystal symmetry. An approximate (isotropic) treatment of cell esds is used for estimating esds involving l.s. planes.

### Fractional atomic coordinates and isotropic or equivalent isotropic displacement parameters (Å^2^)

**Table:**

	*x*	*y*	*z*	*U*_iso_*/*U*_eq_	
Ru1	0.36323 (2)	0.25559 (2)	0.30233 (2)	0.01497 (8)	
Cl1	0.27925 (3)	0.19526 (8)	0.21120 (5)	0.02716 (19)	
Cl2	0.43877 (3)	0.35398 (7)	0.35226 (5)	0.02444 (18)	
N1	0.38319 (10)	0.0907 (2)	0.26624 (14)	0.0209 (6)	
N2	0.35668 (10)	0.4598 (2)	0.38862 (14)	0.0174 (5)	
N3	0.33199 (10)	0.5233 (2)	0.28339 (14)	0.0198 (6)	
C1	0.43550 (12)	0.0483 (3)	0.33159 (18)	0.0259 (8)	
H1	0.455358	0.125580	0.355837	0.031*	
C2	0.46125 (14)	−0.0283 (4)	0.3115 (2)	0.0355 (9)	
H2A	0.495658	−0.042027	0.355392	0.053*	
H2B	0.459579	0.017417	0.275447	0.053*	
H2C	0.444691	−0.108425	0.290984	0.053*	
C3	0.43498 (12)	−0.0165 (3)	0.38758 (18)	0.0234 (7)	
C4	0.46076 (13)	−0.1295 (3)	0.4219 (2)	0.0298 (8)	
H4	0.476461	−0.168616	0.407563	0.036*	
C5	0.46369 (14)	−0.1853 (4)	0.4767 (2)	0.0351 (9)	
H5	0.481190	−0.260705	0.498552	0.042*	
C6	0.44114 (13)	−0.1303 (3)	0.49874 (19)	0.0297 (8)	
H6	0.443424	−0.167247	0.535946	0.036*	
C7	0.41467 (12)	−0.0186 (3)	0.46528 (18)	0.0246 (7)	
H7	0.399250	0.018681	0.480504	0.029*	
C8	0.41055 (11)	0.0395 (3)	0.40913 (17)	0.0195 (7)	
C9	0.38247 (11)	0.1576 (3)	0.37985 (17)	0.0192 (7)	
H9	0.372759	0.188017	0.404817	0.023*	
C10	0.34805 (13)	−0.0197 (3)	0.23194 (19)	0.0290 (8)	
H10A	0.345193	−0.051843	0.266234	0.044*	
H10B	0.360709	−0.084894	0.220549	0.044*	
H10C	0.315705	0.006978	0.187030	0.044*	
C11	0.38039 (15)	0.1501 (3)	0.20776 (19)	0.0315 (8)	
H11A	0.406681	0.211464	0.230071	0.047*	
H11B	0.348547	0.191039	0.171901	0.047*	
H11C	0.384139	0.086122	0.183470	0.047*	
C12	0.34786 (11)	0.4203 (3)	0.32755 (16)	0.0161 (6)	
C13	0.34674 (13)	0.5960 (3)	0.38801 (18)	0.0227 (7)	
H13A	0.376768	0.640104	0.429556	0.027*	
H13B	0.320685	0.608402	0.389721	0.027*	
C14	0.32972 (14)	0.6408 (3)	0.31387 (19)	0.0267 (8)	
H14A	0.295961	0.674526	0.281612	0.032*	
H14B	0.352141	0.704645	0.321270	0.032*	
C15	0.36943 (12)	0.3844 (3)	0.44852 (16)	0.0179 (7)	
C16	0.41955 (13)	0.3761 (3)	0.51418 (18)	0.0235 (7)	
C17	0.43046 (14)	0.3025 (3)	0.57136 (19)	0.0298 (8)	
H17	0.463580	0.295647	0.615705	0.036*	
C18	0.39371 (16)	0.2390 (3)	0.5645 (2)	0.0335 (9)	
C19	0.34442 (15)	0.2522 (3)	0.4989 (2)	0.0304 (8)	
H19	0.319462	0.211225	0.494219	0.036*	
C20	0.33091 (12)	0.3250 (3)	0.43947 (18)	0.0212 (7)	
C21	0.45973 (13)	0.4446 (3)	0.52318 (19)	0.0316 (8)	
H21A	0.491880	0.414900	0.567151	0.047*	
H21B	0.457045	0.533797	0.527643	0.047*	
H21C	0.456104	0.429297	0.480524	0.047*	
C22	0.40732 (18)	0.1573 (4)	0.6276 (2)	0.0503 (12)	
H22A	0.440341	0.178879	0.672911	0.075*	
H22B	0.406526	0.069698	0.616245	0.075*	
H22C	0.383708	0.171613	0.633607	0.075*	
C23	0.27768 (13)	0.3329 (3)	0.3681 (2)	0.0299 (8)	
H23A	0.256838	0.285893	0.372514	0.045*	
H23B	0.274738	0.298087	0.328510	0.045*	
H23C	0.267244	0.419876	0.357329	0.045*	
C24	0.31131 (13)	0.5223 (3)	0.20761 (17)	0.0220 (7)	
C25	0.26105 (13)	0.4902 (3)	0.15098 (19)	0.0271 (8)	
C26	0.24212 (15)	0.4841 (4)	0.0789 (2)	0.0412 (10)	
H26	0.209202	0.458177	0.040854	0.049*	
C27	0.27115 (18)	0.5156 (4)	0.0622 (2)	0.0500 (12)	
C28	0.31984 (17)	0.5575 (4)	0.1182 (2)	0.0446 (11)	
H28	0.338852	0.582460	0.106630	0.054*	
C29	0.34055 (14)	0.5626 (3)	0.19176 (19)	0.0294 (8)	
C30	0.22693 (13)	0.4698 (3)	0.1655 (2)	0.0342 (9)	
H30A	0.245338	0.432243	0.213694	0.051*	
H30B	0.200096	0.414548	0.128166	0.051*	
H30C	0.213420	0.549657	0.163869	0.051*	
C31	0.2495 (2)	0.5069 (6)	−0.0179 (3)	0.087 (2)	
H31A	0.275683	0.486405	−0.017664	0.131*	
H31B	0.234799	0.586665	−0.042432	0.131*	
H31C	0.224272	0.442093	−0.043926	0.131*	
C32	0.39242 (14)	0.6178 (3)	0.2506 (2)	0.0337 (9)	
H32A	0.405493	0.592815	0.299048	0.051*	
H32B	0.390623	0.708490	0.247040	0.051*	
H32C	0.414020	0.587167	0.243167	0.051*	

### Atomic displacement parameters (Å^2^)

**Table:**

	*U*^11^	*U*^22^	*U*^33^	*U*^12^	*U*^13^	*U*^23^
Ru1	0.01431 (13)	0.01479 (14)	0.01431 (13)	−0.00060 (10)	0.00891 (10)	0.00059 (10)
Cl1	0.0164 (4)	0.0262 (4)	0.0285 (4)	−0.0033 (3)	0.0106 (4)	−0.0033 (4)
Cl2	0.0179 (4)	0.0208 (4)	0.0299 (4)	−0.0027 (3)	0.0139 (4)	0.0001 (3)
N1	0.0227 (14)	0.0206 (14)	0.0195 (14)	0.0009 (11)	0.0141 (12)	0.0008 (11)
N2	0.0215 (14)	0.0123 (13)	0.0168 (13)	0.0009 (10)	0.0121 (12)	0.0008 (10)
N3	0.0217 (14)	0.0170 (14)	0.0181 (14)	0.0037 (11)	0.0122 (12)	0.0029 (11)
C1	0.0222 (17)	0.0245 (18)	0.0259 (18)	0.0056 (14)	0.0139 (16)	−0.0023 (14)
C2	0.035 (2)	0.035 (2)	0.042 (2)	0.0024 (17)	0.028 (2)	−0.0011 (17)
C3	0.0200 (17)	0.0206 (18)	0.0221 (17)	−0.0009 (13)	0.0110 (15)	0.0007 (14)
C4	0.0249 (18)	0.0256 (19)	0.030 (2)	0.0017 (15)	0.0149 (16)	0.0021 (16)
C5	0.031 (2)	0.023 (2)	0.035 (2)	0.0027 (16)	0.0157 (18)	0.0066 (17)
C6	0.0279 (19)	0.028 (2)	0.0216 (18)	−0.0021 (15)	0.0115 (16)	0.0076 (15)
C7	0.0250 (18)	0.0217 (18)	0.0238 (18)	−0.0033 (14)	0.0149 (16)	−0.0001 (14)
C8	0.0179 (15)	0.0174 (16)	0.0160 (15)	−0.0042 (12)	0.0083 (14)	−0.0005 (12)
C9	0.0217 (16)	0.0181 (17)	0.0194 (16)	−0.0013 (13)	0.0143 (14)	−0.0031 (13)
C10	0.032 (2)	0.0216 (18)	0.030 (2)	−0.0042 (15)	0.0187 (17)	−0.0067 (15)
C11	0.044 (2)	0.031 (2)	0.030 (2)	0.0041 (17)	0.0288 (19)	0.0025 (16)
C12	0.0116 (14)	0.0185 (16)	0.0162 (15)	−0.0025 (12)	0.0085 (13)	0.0007 (12)
C13	0.0257 (17)	0.0174 (17)	0.0241 (17)	0.0042 (14)	0.0161 (15)	0.0014 (13)
C14	0.035 (2)	0.0172 (18)	0.0313 (19)	0.0064 (15)	0.0233 (17)	0.0026 (14)
C15	0.0233 (16)	0.0145 (16)	0.0142 (15)	0.0014 (13)	0.0117 (14)	−0.0017 (12)
C16	0.0268 (18)	0.0194 (17)	0.0214 (17)	0.0012 (14)	0.0149 (15)	−0.0016 (14)
C17	0.033 (2)	0.0285 (19)	0.0187 (17)	0.0068 (16)	0.0134 (16)	0.0001 (15)
C18	0.053 (2)	0.027 (2)	0.034 (2)	0.0070 (18)	0.034 (2)	0.0039 (16)
C19	0.044 (2)	0.0282 (19)	0.035 (2)	−0.0015 (17)	0.0331 (19)	0.0006 (16)
C20	0.0277 (17)	0.0165 (17)	0.0260 (18)	−0.0011 (14)	0.0206 (16)	−0.0031 (14)
C21	0.0197 (17)	0.032 (2)	0.0253 (19)	−0.0008 (15)	0.0076 (15)	0.0024 (16)
C22	0.073 (3)	0.049 (3)	0.044 (3)	0.015 (2)	0.045 (3)	0.018 (2)
C23	0.0277 (19)	0.030 (2)	0.034 (2)	−0.0045 (15)	0.0220 (17)	−0.0061 (16)
C24	0.0284 (18)	0.0182 (17)	0.0186 (16)	0.0048 (14)	0.0153 (15)	0.0042 (13)
C25	0.0275 (19)	0.0203 (18)	0.0241 (18)	0.0041 (14)	0.0133 (16)	0.0047 (14)
C26	0.038 (2)	0.044 (2)	0.0205 (19)	0.0013 (19)	0.0107 (18)	0.0057 (17)
C27	0.064 (3)	0.057 (3)	0.024 (2)	−0.001 (2)	0.027 (2)	0.002 (2)
C28	0.057 (3)	0.053 (3)	0.035 (2)	−0.001 (2)	0.035 (2)	0.008 (2)
C29	0.034 (2)	0.0265 (19)	0.0279 (19)	0.0015 (16)	0.0206 (17)	0.0053 (15)
C30	0.0260 (19)	0.029 (2)	0.038 (2)	0.0041 (16)	0.0172 (18)	0.0030 (17)
C31	0.099 (5)	0.120 (5)	0.037 (3)	−0.024 (4)	0.043 (3)	−0.010 (3)
C32	0.036 (2)	0.029 (2)	0.039 (2)	0.0002 (16)	0.0264 (19)	0.0064 (17)

### Geometric parameters (Å, º)

**Table:**

Ru1—C9	1.822 (3)	C14—H14B	0.9700
Ru1—C12	2.036 (3)	C15—C20	1.394 (4)
Ru1—N1	2.265 (3)	C15—C16	1.396 (4)
Ru1—Cl1	2.3397 (8)	C16—C17	1.392 (5)
Ru1—Cl2	2.3476 (8)	C16—C21	1.492 (5)
N1—C11	1.483 (4)	C17—C18	1.386 (5)
N1—C10	1.497 (4)	C17—H17	0.9300
N1—C1	1.503 (4)	C18—C19	1.384 (5)
N2—C12	1.349 (4)	C18—C22	1.519 (5)
N2—C15	1.431 (4)	C19—C20	1.397 (5)
N2—C13	1.480 (4)	C19—H19	0.9300
N3—C12	1.354 (4)	C20—C23	1.494 (5)
N3—C24	1.442 (4)	C21—H21A	0.9600
N3—C14	1.471 (4)	C21—H21B	0.9600
C1—C3	1.524 (4)	C21—H21C	0.9600
C1—C2	1.528 (4)	C22—H22A	0.9600
C1—H1	0.9800	C22—H22B	0.9600
C2—H2A	0.9600	C22—H22C	0.9600
C2—H2B	0.9600	C23—H23A	0.9600
C2—H2C	0.9600	C23—H23B	0.9600
C3—C4	1.394 (5)	C23—H23C	0.9600
C3—C8	1.411 (4)	C24—C25	1.394 (5)
C4—C5	1.385 (5)	C24—C29	1.399 (5)
C4—H4	0.9300	C25—C26	1.383 (5)
C5—C6	1.357 (5)	C25—C30	1.493 (5)
C5—H5	0.9300	C26—C27	1.383 (6)
C6—C7	1.386 (5)	C26—H26	0.9300
C6—H6	0.9300	C27—C28	1.387 (6)
C7—C8	1.401 (4)	C27—C31	1.529 (6)
C7—H7	0.9300	C28—C29	1.393 (5)
C8—C9	1.456 (4)	C28—H28	0.9300
C9—H9	0.9300	C29—C32	1.514 (5)
C10—H10A	0.9600	C30—H30A	0.9600
C10—H10B	0.9600	C30—H30B	0.9600
C10—H10C	0.9600	C30—H30C	0.9600
C11—H11A	0.9600	C31—H31A	0.9600
C11—H11B	0.9600	C31—H31B	0.9600
C11—H11C	0.9600	C31—H31C	0.9600
C13—C14	1.528 (4)	C32—H32A	0.9600
C13—H13A	0.9700	C32—H32B	0.9600
C13—H13B	0.9700	C32—H32C	0.9600
C14—H14A	0.9700		
			
C9—Ru1—C12	99.91 (12)	N3—C14—H14A	111.3
C9—Ru1—N1	87.49 (12)	C13—C14—H14A	111.3
C12—Ru1—N1	171.53 (10)	N3—C14—H14B	111.3
C9—Ru1—Cl1	100.30 (10)	C13—C14—H14B	111.3
C12—Ru1—Cl1	94.51 (8)	H14A—C14—H14B	109.2
N1—Ru1—Cl1	88.15 (7)	C20—C15—C16	122.7 (3)
C9—Ru1—Cl2	101.93 (10)	C20—C15—N2	118.4 (3)
C12—Ru1—Cl2	85.25 (8)	C16—C15—N2	118.9 (3)
N1—Ru1—Cl2	89.21 (7)	C17—C16—C15	117.2 (3)
Cl1—Ru1—Cl2	157.47 (3)	C17—C16—C21	121.3 (3)
C11—N1—C10	107.8 (3)	C15—C16—C21	121.5 (3)
C11—N1—C1	111.7 (3)	C18—C17—C16	122.3 (3)
C10—N1—C1	110.4 (3)	C18—C17—H17	118.9
C11—N1—Ru1	101.59 (19)	C16—C17—H17	118.9
C10—N1—Ru1	116.52 (19)	C19—C18—C17	118.4 (3)
C1—N1—Ru1	108.62 (19)	C19—C18—C22	121.0 (4)
C12—N2—C15	127.8 (3)	C17—C18—C22	120.7 (4)
C12—N2—C13	114.2 (2)	C18—C19—C20	122.2 (3)
C15—N2—C13	117.7 (2)	C18—C19—H19	118.9
C12—N3—C24	125.5 (3)	C20—C19—H19	118.9
C12—N3—C14	114.8 (2)	C15—C20—C19	117.2 (3)
C24—N3—C14	119.1 (2)	C15—C20—C23	121.7 (3)
N1—C1—C3	108.6 (3)	C19—C20—C23	121.1 (3)
N1—C1—C2	114.8 (3)	C16—C21—H21A	109.5
C3—C1—C2	114.1 (3)	C16—C21—H21B	109.5
N1—C1—H1	106.2	H21A—C21—H21B	109.5
C3—C1—H1	106.2	C16—C21—H21C	109.5
C2—C1—H1	106.2	H21A—C21—H21C	109.5
C1—C2—H2A	109.5	H21B—C21—H21C	109.5
C1—C2—H2B	109.5	C18—C22—H22A	109.5
H2A—C2—H2B	109.5	C18—C22—H22B	109.5
C1—C2—H2C	109.5	H22A—C22—H22B	109.5
H2A—C2—H2C	109.5	C18—C22—H22C	109.5
H2B—C2—H2C	109.5	H22A—C22—H22C	109.5
C4—C3—C8	118.0 (3)	H22B—C22—H22C	109.5
C4—C3—C1	121.0 (3)	C20—C23—H23A	109.5
C8—C3—C1	120.9 (3)	C20—C23—H23B	109.5
C5—C4—C3	121.9 (3)	H23A—C23—H23B	109.5
C5—C4—H4	119.0	C20—C23—H23C	109.5
C3—C4—H4	119.0	H23A—C23—H23C	109.5
C6—C5—C4	120.3 (3)	H23B—C23—H23C	109.5
C6—C5—H5	119.9	C25—C24—C29	121.3 (3)
C4—C5—H5	119.9	C25—C24—N3	118.7 (3)
C5—C6—C7	119.4 (3)	C29—C24—N3	119.8 (3)
C5—C6—H6	120.3	C26—C25—C24	118.2 (3)
C7—C6—H6	120.3	C26—C25—C30	119.6 (3)
C6—C7—C8	121.7 (3)	C24—C25—C30	122.0 (3)
C6—C7—H7	119.1	C27—C26—C25	121.3 (4)
C8—C7—H7	119.1	C27—C26—H26	119.3
C7—C8—C3	118.7 (3)	C25—C26—H26	119.3
C7—C8—C9	116.0 (3)	C26—C27—C28	119.7 (4)
C3—C8—C9	125.3 (3)	C26—C27—C31	120.3 (4)
C8—C9—Ru1	130.6 (2)	C28—C27—C31	120.0 (4)
C8—C9—H9	114.7	C27—C28—C29	120.6 (4)
Ru1—C9—H9	114.7	C27—C28—H28	119.7
N1—C10—H10A	109.5	C29—C28—H28	119.7
N1—C10—H10B	109.5	C28—C29—C24	118.3 (3)
H10A—C10—H10B	109.5	C28—C29—C32	119.2 (3)
N1—C10—H10C	109.5	C24—C29—C32	122.4 (3)
H10A—C10—H10C	109.5	C25—C30—H30A	109.5
H10B—C10—H10C	109.5	C25—C30—H30B	109.5
N1—C11—H11A	109.5	H30A—C30—H30B	109.5
N1—C11—H11B	109.5	C25—C30—H30C	109.5
H11A—C11—H11B	109.5	H30A—C30—H30C	109.5
N1—C11—H11C	109.5	H30B—C30—H30C	109.5
H11A—C11—H11C	109.5	C27—C31—H31A	109.5
H11B—C11—H11C	109.5	C27—C31—H31B	109.5
N2—C12—N3	106.2 (3)	H31A—C31—H31B	109.5
N2—C12—Ru1	132.6 (2)	C27—C31—H31C	109.5
N3—C12—Ru1	120.5 (2)	H31A—C31—H31C	109.5
N2—C13—C14	102.6 (2)	H31B—C31—H31C	109.5
N2—C13—H13A	111.2	C29—C32—H32A	109.5
C14—C13—H13A	111.2	C29—C32—H32B	109.5
N2—C13—H13B	111.2	H32A—C32—H32B	109.5
C14—C13—H13B	111.2	C29—C32—H32C	109.5
H13A—C13—H13B	109.2	H32A—C32—H32C	109.5
N3—C14—C13	102.2 (2)	H32B—C32—H32C	109.5
			
C11—N1—C1—C3	−177.6 (3)	C12—N2—C15—C20	−82.6 (4)
C10—N1—C1—C3	−57.7 (3)	C13—N2—C15—C20	90.7 (3)
Ru1—N1—C1—C3	71.2 (3)	C12—N2—C15—C16	99.2 (4)
C11—N1—C1—C2	−48.4 (4)	C13—N2—C15—C16	−87.6 (3)
C10—N1—C1—C2	71.4 (3)	C20—C15—C16—C17	1.5 (5)
Ru1—N1—C1—C2	−159.7 (2)	N2—C15—C16—C17	179.6 (3)
N1—C1—C3—C4	135.9 (3)	C20—C15—C16—C21	−177.7 (3)
C2—C1—C3—C4	6.3 (5)	N2—C15—C16—C21	0.4 (5)
N1—C1—C3—C8	−47.5 (4)	C15—C16—C17—C18	0.1 (5)
C2—C1—C3—C8	−177.0 (3)	C21—C16—C17—C18	179.4 (3)
C8—C3—C4—C5	−1.4 (5)	C16—C17—C18—C19	−1.4 (5)
C1—C3—C4—C5	175.4 (3)	C16—C17—C18—C22	178.6 (3)
C3—C4—C5—C6	0.0 (6)	C17—C18—C19—C20	1.2 (5)
C4—C5—C6—C7	0.7 (5)	C22—C18—C19—C20	−178.9 (3)
C5—C6—C7—C8	−0.1 (5)	C16—C15—C20—C19	−1.7 (5)
C6—C7—C8—C3	−1.3 (5)	N2—C15—C20—C19	−179.8 (3)
C6—C7—C8—C9	−178.9 (3)	C16—C15—C20—C23	−178.7 (3)
C4—C3—C8—C7	2.0 (5)	N2—C15—C20—C23	3.2 (4)
C1—C3—C8—C7	−174.8 (3)	C18—C19—C20—C15	0.3 (5)
C4—C3—C8—C9	179.3 (3)	C18—C19—C20—C23	177.3 (3)
C1—C3—C8—C9	2.5 (5)	C12—N3—C24—C25	80.9 (4)
C7—C8—C9—Ru1	−173.7 (2)	C14—N3—C24—C25	−89.7 (4)
C3—C8—C9—Ru1	8.9 (5)	C12—N3—C24—C29	−104.1 (4)
C12—Ru1—C9—C8	−162.3 (3)	C14—N3—C24—C29	85.4 (4)
N1—Ru1—C9—C8	13.6 (3)	C29—C24—C25—C26	7.8 (5)
Cl1—Ru1—C9—C8	101.3 (3)	N3—C24—C25—C26	−177.2 (3)
Cl2—Ru1—C9—C8	−75.1 (3)	C29—C24—C25—C30	−168.6 (3)
C15—N2—C12—N3	173.5 (3)	N3—C24—C25—C30	6.3 (5)
C13—N2—C12—N3	0.0 (3)	C24—C25—C26—C27	−3.6 (6)
C15—N2—C12—Ru1	−16.5 (5)	C30—C25—C26—C27	173.0 (4)
C13—N2—C12—Ru1	170.1 (2)	C25—C26—C27—C28	−1.7 (7)
C24—N3—C12—N2	−170.4 (3)	C25—C26—C27—C31	179.4 (4)
C14—N3—C12—N2	0.5 (4)	C26—C27—C28—C29	3.0 (7)
C24—N3—C12—Ru1	18.1 (4)	C31—C27—C28—C29	−178.2 (4)
C14—N3—C12—Ru1	−171.0 (2)	C27—C28—C29—C24	1.1 (6)
C12—N2—C13—C14	−0.4 (3)	C27—C28—C29—C32	−175.5 (4)
C15—N2—C13—C14	−174.6 (3)	C25—C24—C29—C28	−6.6 (5)
C12—N3—C14—C13	−0.8 (4)	N3—C24—C29—C28	178.5 (3)
C24—N3—C14—C13	170.8 (3)	C25—C24—C29—C32	169.9 (3)
N2—C13—C14—N3	0.7 (3)	N3—C24—C29—C32	−5.0 (5)

### Hydrogen-bond geometry (Å, º)

**Table:**

*D*—H···*A*	*D*—H	H···*A*	*D*···*A*	*D*—H···*A*
C1—H1···Cl2	0.98	2.47	3.258 (3)	137
C10—H10*C*···Cl1	0.96	2.65	3.136 (4)	112
C11—H11*A*···Cl2	0.96	2.78	3.381 (4)	121
C13—H13*B*···Cl1^i^	0.97	2.82	3.578 (3)	135
C14—H14*A*···Cl1^i^	0.97	2.82	3.576 (4)	135
C9—H9···*Cg*4	0.93	2.61	3.481 (4)	157

Symmetry code: (i) −*x*+1/2, *y*+1/2, −*z*+1/2.
